# Mold infestation and aflatoxins production in traditionally processed spices and aromatic herbs powder mostly used in West Africa

**DOI:** 10.1002/fsn3.579

**Published:** 2018-02-02

**Authors:** Djinoumi Olivia Opportune Akpo‐Djènontin, Fernand Gbaguidi, Mohamed M. Soumanou, Victor B. Anihouvi

**Affiliations:** ^1^ Laboratory of Science School of Nutrition, Science and Food Technology Faculty of Agronomic Sciences University of Abomey‐Calavi Cotonou Benin; ^2^ Faculty of Sciences and Techniques University of Abomey‐Calavi Cotonou Benin; ^3^ Laboratory of Study and Research in Applied Chemistry Cotonou Benin

**Keywords:** dried products, food safety, fungi, mycotoxins, occurrence, powder

## Abstract

Mold infestation and occurrence of aflatoxins were investigated in 66 samples of dried spices and aromatic herbs powder (SAH) as commercialized in Benin and its neighboring countries. The samples were randomly collected from markets, supermarkets, and processing sites. Mold counts were enumerated according to standard method and aflatoxins levels were assessed using high‐performance liquid chromatography coupled with fluorescence detection (HPLC‐FLD). The results revealed that mold counts of samples ranged between 2.62 and 4.34 LogCFU/g. Aflatoxin B_1_ contents were between 0.46 μg/kg and 84.84 μg/kg with 40% of samples exceeding the recommended limit of 5 μg/kg. Aflatoxins G_1_ and G_2_ levels were low in general with means values varying from 0.24 to 8.56 μg/kg, and 0.11 to 3.68 μg/kg, respectively. Fifty‐two percent (52%) of samples analyzed contained total aflatoxins levels lower than the stipulated limit of 10 μg/kg, whereas 92% of them were contaminated at various levels with one type aflatoxin, B_1_ or B_2_, G_1_ or G_2_. This study provides the first information about the occurrence of aflatoxins in the common spices used in West Africa.

## 1 INTRODUCTION

Spices and aromatic herbs (SAH) used in West Africa region were vegetables mostly produced under tropical climate, materialized by high humidity and rainfall conditions which favored the production of mycotoxins Martins et al (2001). Mycotoxins are toxic components from secondary metabolism of certain species of fungi. The main documented mycotoxins were deoxynivalenol (produced by *Fusarium graminearum*), zearalenon (by *Fusarium culmorum or Fusarium crookwellense*), furninosin (by *Fusarium moniliform or, Fusarium proliferatum*), ochratoxin (by *Penicillium verrucosum*), and aflatoxins (by *Aspergillus flavus*) (Adibian, 2016; Miller, 1996). Factors implicated in the growth of these fungi in foodstuffs are those related to the environment in which they develop (pH, composition of the food, or water activity) and other extrinsic factors such as ambient humidity, storage temperature, and microbial competition (Al‐juraifani, 2011; Mably et al., 2005). The most dangerous among the 400 types of mycotoxins known are aflatoxins. Aflatoxins can occur in foods, such as groundnuts, maize, rice, spices, and other dried products, because of fungal contamination before and after harvest. In general, 18 types of aflatoxins were observed in foodstuffs with four most frequently (B_1_, B_2_, G_1_ and G_2_) (Arshad & Muhammad, 2012; Naphaporn et al 2015) and more produced by *A flavus*, and in a lesser extent by *Aspergillus parasiticus* and *Aspergillus nominus* (Miller, 1996). Spices and aromatic herbs would be inappropriate for consumption after being contaminated by mycotoxins produced by fungi during growth, harvesting, postharvest, or transportation (Akpo‐Djènontin et al., 2016; Tajkarimi et al 2011). In addition, aflatoxins contamination rate can also be enhanced by the inadequate conditions of drying (sun drying on the floor, on the sidewalks, extended drying times) and storage practices (unhygienic condition and an instable environmental conditions) (Akpo‐Djènontin et al., 2016; Nakai et al., 2008). Fungal contamination of dried products leading to the production of aflatoxins has been reported worldwide but it is more expressed in the developing countries (Matthews, 2005). There are more than 5 billion people in developing countries at risk of chronic exposure to aflatoxins through contaminated foods van Egmond et al (2007). Since aflatoxins are known to be genotoxic and carcinogenic, exposure through food should be kept as low as possible (OMS, 2002; Roy & Chourasia, 1990). Therefore, the International Agency for Research on Cancer (IARC) has classified since 1987 aflatoxins in carcinogen group of chemical components. The aflatoxin B_1_ is more harmful and represents the most critical indicator of food toxicity Wacoo et al (2014). Risks associated with mycotoxins depend on both hazard and exposure. While risk could be same around the world, exposure is not the same, because of differences in levels of contamination and dietary habits in various parts of the world. For example, in a country, where the maize consumption is approximately 15 g/capita/day, a legal limit of 8 mg/kg would suffice to prevent fumonisin effect. However, in another country, where the maize consumption is about 125 g/capita/day, a legal limit of 1 mg/kg would be required to reach the same level of protection (Van Egmond et al., 2007). According to Codex Alimentarius (2009), the recommended limit of total aflatoxins is 10 μg/kg for nuts, almonds, and many foodstuffs. With regard to SAH, legal limits of 10 μg/kg and 5 μg/kg are stipulated for total aflatoxins and aflatoxin B_1_, respectively (EC, 2006). Nowadays the level of consumption of SAH is much raising. About 80% of the population in developing countries consume spices and aromatic herbs (Van Andel, 2006). In Benin, its daily consumption is estimated to 5 g/capita (FAOSTAT, 2012). Thus, the aim of this study is to investigate the safety status of SAH processed and consumed in West Africa region in regard to possible contamination by aflatoxins.

## 2 MATERIALS AND METHODS

### 2.1 Samples collection

**Figure 1:**
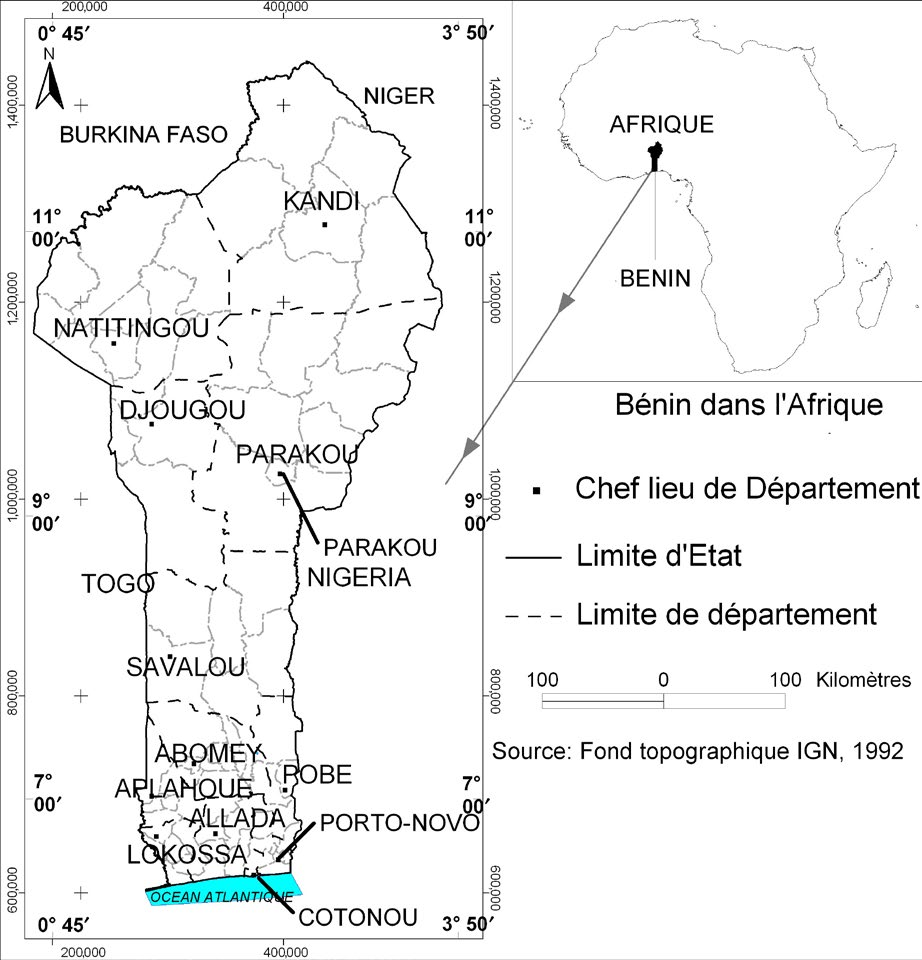
Map showing the sampling zones

A total of 66 samples of SAH were randomly purchased from retailers in markets, from supermarkets and processing sites at a rate of 22 samples per sampling zone and six per type of samples (Table 1). The samples were collected from the cosmopolitan cities of Cotonou, Porto‐Novo, and Parakou (Figure 1). The sampling zones were selected based on a previous work (Akpo‐Djènontin et al., 2016) which revealed the main processing and consumption areas of SAH in Benin. The samples consisted of single herbs and SAH powders processed within 3 months and more than 3 months old.

**Table 1:** Samples of spices and aromatic herb powders as commercialized in Benin included in this study

SAH powders	Composition of mixtures (Variants)	Codes of samples	Number of samples collected
Mixture of 3 SAH	Garlic–ginger–pepper	GaGiP	6
	Garlic–chili–pepper	GaCP	6
	Garlic–ginger–laurel	GaGiL	6
Mixture of 7 SAH	Garlic–ginger–nutmeg–dill–chili–pepper–laurel	GaGiLPCND	6
	Garlic–ginger–nutmeg–clove chili–pepper–laurel	GaGiLPCNCl	6
	Ginger–rosemary–thyme–clove–laurel–nutmeg–Cinnamon	GiRTClLNCi	6
Mixture of 12 SAH	Ginger–laurel–pepper–cumin–thyme–rosemary–cinnamon–anise–curry	GiLPCmTRCiACu*	6
	Garlic–thyme–curry–pepper–laurel–basil–nutmeg–ginger–clove	GaTCu*PLBNGiCl	6
Mixture for tchachanga	Dry chili–nutmeg–cake of peanut–thyme–salt, maggi	CTch	6
Herbs	Dry basil Dry laurel	DB DL	6 6

Cu*: blend of spices with variable composition, but composed at least of four spices including curcuma, cumin, coriander, and fenugreek**.**

### 2.2 Enumeration of molds

For all samples, 10 g was weighed aseptically in a sterile stomacher bag and suspended in 90 ml of sterile diluents containing 0.1% peptone (Oxoid L 37, Basingstoke, Hampshire, England) and 0.8% sodium chloride (NaCl) with pH adjusted to 7.2. The mixture was macerated for 2 min using a stomacher (Lab Blender, Model 400). One (1) ml of the homogenate was serially diluted and used for enumeration of molds on petri plates using chloramphenicol glucose agar (Biokar diagnostics‐zac de ther‐allone‐F60000 Beauvais). The plates were incubated at 25°C for 3–5 days (ISO 7954: 1988).

### 2.3 Detection and quantification of aflatoxins

#### 2.3.1 Samples preparation

##### Extraction

Approximately 25 g of SAH powder was mixed with 125 ml of methanol: deionized water (80:20), 62.5 ml of hexane, and 2.5 g of NaCl. The mixture was shaken for 30 min and filtered through Whatman N°1 filter paper.

##### Purification

Fifteen mL of the resulting extract was diluted with 86 ml of phosphate‐buffered saline solution (PBS; pH 7.2) and filtrated. Eleven (11) ml of this solution was applied to an immunoaffinity column (Easi‐extract aflatoxin) containing specific antibodies of aflatoxins B_1_, B_2_, G_1_, and G_2_ at a flow rate of 1–2 drops/s, and the column washed again with 10 ml of PBS. The toxins were then eluted with 1 ml of methanol at a flow rate of 0.3 ml/min. After 5 min approximately, a second portion of 500 μl of methanol was applied followed by another one with 3.5 ml of deionized water to ensure complete disruption of the antibody‐toxin bond.

#### 2.3.2 Chromatographic conditions

Aflatoxins quantification was performed according to ISO 16050 (2003) using the Dionex HPLC system (Dionex, Ultimate 3000, Netherlands) with a fluorescence detector (Dionex 2000, Netherland), a pump (DGP 3600 A), a C18 column (4.6 mm × 250 mm × 5 μm id, Dionex, Netherlands) with a post derivatisation column and a 20 μl injector loop (WPS 3000 TSL, Dionex, Netherlands). The analysis was carried out isocratically at a flow rate of 1 ml/min using methanol/deionized water solution (80:20) as mobile phase. A volume of 10 μl of extract was injected and aflatoxins B_1_, B_2_, G_1_ and G_2_ were detected by their retention times and quantified using the peaks areas compared to standard. The excitation and emission wavelengths of 365 and 435 nm were set during the analysis, respectively.

### 2.4 Determination of water activity

Water activity (*a*_w_) of samples was measured with a thermo hygrometer (Hygrolab model 3 w/o 39746, Rotronic), according to the method described by Anihouvi et al. 2006.

### 2.5 Statistical analysis

The data were analyzed using Statistica software (version 7.1). One‐way analysis of variance (ANOVA) was applied to compare means of aflatoxins concentrations and fungal loads. Significance of differences was established at *p* < .05. Correlations between variables (duration of preservation, water activity, fungal loads, total aflatoxins, and sampling places) were assessed with principal component analysis (PCA) using XLSTAT software (version 2011, Addinsoft, Paris, France).

## 3 RESULTS AND DISCUSSION

### 3.1 Microbiological status of mixtures of spices and herbs powder analyzed

The results showed that mold contamination occurred in all the samples investigated (Table 2). The averages fungal counts varied from 2.71 to 4.34 LogCFU/g, 2.62 to 3.32 LogCFU/g, and 2.63 to 3.68 LogCFU/g for market samples, supermarkets samples, and processing sites samples, respectively. The highest fungal load (4.34 LogCFU/g) was found in the mixture collected from markets and formulated with seven different spices. However, it was noticed that the fungal load of samples did not increase according to the number of individual spices or aromatic herbs mixed. Mold counts ranging between 1.85 and 5.6 LogCFU/g, and between 3.4 and 6.7 LogCFU/g have been reported in various spices (Koci‐Tanackov et al (2007); Salari et al (2012). In the same way, Zinedine et al. (2006) have reported that, samples of pepper, paprika, and spice blends were frequently contaminated by various molds including *Eurotium herbariorum* and *Aspergillus versicolor*. With the exceptions of samples GaGiLPCND collected from markets and GaTCu*PLBNGiCl sampled from processing sites, there are no significant difference (*p* > .05) between molds counts of the same blends of spices collected from the different sampling places (Table 2). In addition, the fungal loads of most of samples (60%) analyzed were lower than the acceptable limit of 3–4 LogCFU/g stipulated by European Spice Association (ESA, 2004) and the International Commission on Microbiological Specifications for Foods ICMSF (2011). The results also showed that fungal counts of samples with shelf life more than 3 months were significantly (*p* < .05) higher than those of samples with shelf life lower than 3 months (data not shown). The variation in fungal loads of SAH powder samples could be a consequence of processing practices, long preservation period, storage room conditions, and status of each raw spice and aromatic herb used as previously reported (Koci‐Tanackov et al., 2007; Matthews & Jack,^2011^). The high prevalence of molds in dried foods and their by‐products is presumably due to low water activity (*a*_w_) required for their growth (Naphaporn et al., 2015).

**Table 2:** Fungal loads in SAH powder samples collected from different selling places

Samples of SAH powder (*n* = 66)	Fungal loads (logCFU/g)		
	Markets	Supermarkets	Processing sites
GaGiP	2.71 ± 0.13^a^	3.11 ± 0.12^a^	2.63 ± 0.13^a^
GaCP	2.94 ± 0.16^a^	2.62 ± 0.16^a^	2.95 ± 0.09^a^
GaGiL	3.08 ± 0. 5^a^	2.89 ± 0.09^a^	2.68 ± 0.61^a^
GaGiLPCND	4.34 ± 0.61^b^	3.32 ± 0.42^a^	3.58 ± 0.54^a^
GaGiLPCNCl	1.86 ± 0.09^a^	3.15 ± 0.084^a^	3.36 ± 0.095^a^
GiRTClLNCi	3.43 ± 0.25^a^	3.08 ± 0.25^a^	2.93 ± 0.25^a^
GiLPCmTRCiACu*	2.94 ± 0.23^a^	2.68 ± 0.23^a^	3.32 ± 0.23^a^
GaTCu*PLBNGiCl	3.11 ± 0.12^a^	2.9 ± 0.13^a^	2.63 ± 0.13^a^
CTch	2.62 ± 0.08^a^	2.71 ± 0.08^a^	2.75 ± 0.08^a^
DB	1.44 ± 0.05^a^	1.64 ± 0.01^a^	2.07 ± 0.07^a^
DL	1.34 ± 0.04^a^	0.93 ± 0.01^a^	1.21 ± 0.06^a^

*n*, number of samples analyzed.

^a,b^Means with different letters on each row are significantly different (*p *< .05).

### 3.2 Aflatoxins occurrence in spices and aromatic herbs samples investigated

The results obtained showed that the samples investigated were not exempt of mycotoxins. Most of samples (92%) are at least contaminated with one type of aflatoxin, B_1_ or B_2_, G_1_ or G_2,_ and 84% of them contaminated by aflatoxin B1 (AFB1) (Table 3). The incidence of aflatoxins noted for some of the samples analyzed (40%) exceed the permissible level of 5 μg/kg stipulated by European Commission (EC, 2006) concerning the type B1. Similarly, Azzoune et al. (2015) reported that 63.9% of 36 samples of spices investigated in Algeria, contained AFB_1_ at levels ranging from 0.10 to 26.50 μg/kg. Work carried out by Cavit (2005) on various spices including paprika, chili powder, and ground black pepper samples revealed that these spices were contaminated by aflatoxin B_1_ with levels ranging between 0.5–116.4, 1.6–80.4, and 0.3‐1.2 μg/kg, respectively. In addition, 30% paprika and chili powder samples contained AFB_1_ at levels above the regulatory limit of 5 μg/kg recommended by the European Commission. These previous findings are similar to the results obtained from this study and confirmed that spices could be a potential vehicle of food‐borne contaminants. The aflatoxin type B1 is the most dangerous, classified as a carcinogen agent by the International Agency for Research on Cancer (IARC, 1993). The high extent of aflatoxin B1 contamination in some of the samples investigated (up to 84.9 μg/kg) is still significant and show that there is a need for training and sensitization to improve handling and processing procedures of SAH commercialized in West Africa countries. The high content in aflatoxin B1 is certainly due to the unhygienic conditions during processing and handling of these blend of spices, and the composition of the blends as well (Arshad & Muhammad, 2012; Salari et al., 2012). Similar observations were made by Akpo‐Djènontin et al. (2016) who reported as potential causes of SAH contamination, the drying conditions, the contact of SAH with animals, the dust, and climatic risks. In addition, the mechanical damages of SAH and other injuries during plant growing, harvest or agricultural practices could induce molds contamination and lead to the production of aflatoxins (CAC, 2003; Codex Alimentarus, 2015). The production of aflatoxins in SAH may also depend on the ability of each SAH to be used as substrate (Llewellyn, Burkett, & Eadie, 1981). For example, ginger and rosemary leaves were substantial mycotoxins producing‐substrates; mustard, caraway seed, and celery seed were judged as intermediate‐producing substrates. Absolute antimycotic substrates were cinnamon and clove. Antiaflatoxigenic substrates were thyme and oregano while mustard may be antimycotic (Llewellyn et al., 1981). The packaging methods and dust can accentuate the production of mycotoxins and consequently invaded SAH during processing and storage (Ndaw, 2015). In this respect, sorting of SAH that took place before drying or milling constitutes in this fact, a critical point to be taken into account during processing of SAH to reduce mycotoxins incidence (Akpo‐Djènontin et al., 2016; Bankolé & Adebanjo, 2003). Consequently, reducing the fungal contamination of SAH is the most promising strategy for reducing AFB1. Furthermore, the results revealed the presence of aflatoxins G1 and G2 in the samples investigated with maximum levels of 8.56 μg/kg and 3.7 μg/kg, respectively, and this occurred in 32% and 28% of samples, respectively. Regarding the sum of the four aflatoxins (B1 + B2 + G1 + G2), the samples collected from markets were more contaminated (0.62–99.3 μg/kg) when compared with those collected from processing sites (0–29.3 μg/kg) and those from supermarkets (0–21.2 μg/kg) (Table 4). Fifty‐two percent (52%) of the samples analyzed showed total aflatoxins contents lower than the recommended limit of 10 μg/kg. The 48% more contaminated samples by total aflatoxins (>10 μg/kg) were distributed as: 8% from the processing sites, 24% from the markets, and 16% from the supermarkets. Thus, despite a lack of information on food poisoning due to aflatoxins, there is a potential for sporadic aflatoxin poisoning related to the consumption of SAH powder commercialized in Benin and other countries of West Africa region.

**Table 3:** Aflatoxins contents in samples of SAH powder according to the duration of preservation

Samples (*n* = 60)	Shelf life (months)	Aflatoxins (μg/kg)			
		B1	B2	G1	G2
GaGiP	>3	6.21 ± 0.04^a^	0.81 ± 0.03^a^	3.4 ± 0.01^a^	1.72 ± 0.03^a^
	<3	2.98 ± 0.04^b^	0.47 ± 0.04^b^	2.18 ± 0.03^b^	ND
GaCP	>3	84.84 ± 0.03^a^	14.11 ± 0.05^a^	0.42 ± 0.04^b^	ND
	<3	1.84 ± 0.08^b^	0.38 ± 0.02^b^	ND	ND
GaGiL	>3	24.06 ± 0.11^a^	3.32 ± 0.01^a^	8.56 ± 0.01^a^	1.51 ± 0.04^a^
	<3	4.84 ± 0.0 3^b^	0.46 ± 0.1^b^	5.16 ± 0.04^b^	1.06 ± 0.01^b^
GaGiLPCND	>3	28.31 ± 0.07^a^	5.71 ± 0.01^a^	2 ± 0.01^a^	3.68 ± 0.08^a^
	<3	11.34 ± 0.09^b^	0.86 ± 0.03^b^	ND	ND
GaGiLPCNCl	>3	ND	0.06 ± 0.01^b^	0.24 ± 0.06^b^	ND
	<3	1.84 ± 0.07^a^	0.36 ± 0.04^a^	1.99 ± 0.04^a^	0.49 ± 0.03^a^
GiRTClLNCi	>3	8.86 ± 0.04^a^	0.9 ± 0.03^b^	8.28 ± 0.06^a^	0.83 ± 0.04^b^
	<3	4.65 ± 0.08^b^	3.81 ± 0.03^a^	3.99 ± 0.04^b^	ND
GiLPCmTRCiACu*	>3	2.82 ± 0.03^a^	0.43 ± 0.06^a^	ND	ND
	<3	0.66 ± 0.08^b^	ND	0.53 ± 0.04^a^	ND
GaTCuPLBNGiCl	>3	0.46 ± 0.05^b^	0.24 ± 0.06^a^	0.84 ± 0.06^a^	0.11 ± 0.01^a^
	<3	0.66 ± 0.06^a^	0.09 ± 0.01^b^	ND	ND
CTch	>3	24.22 ± 0.01^a^	6.26 ± 0.01^a^	1.48 ± 0.03^a^	0.21 ± 0.01^a^
	<3	17.37 ± 0.04^b^	3.43 ± 0.03^b^	0. 17 ± 0.04^b^	ND
Herbs	>3	<LOQ	<LOD	<LOD	0.62
	<3	<LOQ	<LOD	<LOD	<LOD

*n* = *n*umber of samples analyzed; LOD, limit of detection; LOQ, limit of quantification.

^a‐c^Means with different letters in the same column are significantly different (*p* < .05).

**Table 4:** Total aflatoxins means (sum of B_1_, B_2_, G_1_ and G_2_) per sampling sites

Samples of SAH (*n* = 6)	Total aflatoxins (μg/kg)		
	Markets	Supermarkets	Processing sites
GaGiP	12.14 ± 2.37^A^	—	5.63 ± 1.19^B^
GaCP	99.37 ± 45.31^A^	2.23 ± 1.03^C^	15.6 ± 5.66^B^
GaGiL	37.45 ± 10.24^A^	11.58 ± 2.47^B^	—
GaGiLPCND	39.7 ± 12.35^A^	12.19 ± 7.41^B^	—
GaGiLPCNCl	0.29 ± 0.13^B^	—	4.69 ± 0.78^A^
GiRTClLNCi	18.87 ± 4.45^A^	12.46 ± 0.44^B^	5.05 ± 0.93^C^
GiLPCmTRCiACu*	3.25 ± 1.68^A^	3.25 ± 1.69 A	1.19 ± 0.09^B^
GaTCu*PLBNGiCl	—	1.65v0.32 A	0.75 ± 0.40^B^
CTch	32.15 ± 11.08^A^	21.02 ± 9.16^A^	29.3 ± 11.24^B^
Herbs	0.62 ± 0.0^A^	<LOQ	<LOQ

*n*, number of samples analyzed per type of SAH and according to the sampling place; LOQ, limit of quantification.

^A‐C^Means with different letters on each row are significantly different (*p* < .05).

### 3.3 Water activity of samples analyzed

The water activity (*a*_w_) values varied from one sample to another and ranged between 0.3 and 0.67. The *a*_w_ was analyzed in each sample to establish whether there was a link between *a*_w_ and total aflatoxins of the samples. If this relationship did exist, the *a*_w_ determination, which is quite rapid, could be used as a simple test to assess possible aflatoxin content. The results showed that there is no direct relationship between *a*_w_ and aflatoxin content, as seen from Table 5. However, according to Gallo et al. (2016), temperature and water activity are the two key determinants influencing both the rate of fungal spoilage and aflatoxin production. In this regard, maximum fungal growth and aflatoxin production were noticed at 28°C and 0.96 *a*_w_, whereas good fungal growth, but a very low aflatoxin B_1_ production occurred at 37°C with 0.93–0.99 *a*_w_ (Gallo et al. 2016). Results of this study were not in accordance with growing conditions of molds and aflatoxin B_1_ occurrence stipulated above. Thus, the presence of aflatoxins may not be only explained by the water activity as reported by Ramesh and Jayagoudar (2013). Similar relationship was established between mold population growth and water activity (Nyugen, 2007), or moisture content (Al‐juraifani, 2011; Bircan et al., 2008) with temperature ranging between 20°C and 45°C (AFSSA, 2009).

**Table 5:** Water activity and total aflatoxins contents of samples investigated

Samples of SAH (*n* = 6)	Water activity	Total aflatoxins (μg/kg)
GaGiP	0.48 ± 0.06^b^	8.88 ± 3.74^c^
GaCP	0.44 ± 0.07^b^	39.06 ± 47.10^a^
GaGiL	0.46 ± 0.02^b^	24.45 ± 14.96^b^
GaGiLPCND	0.3 ± 0.1^c^	25.94 ± 15.86^b^
GaGiLPCNCl	0.48 ± 0.08^b^	2.47 ± 2.51^d^
GiRTCLNCi	0.49 ± 0.1^b^	10.69 ± 5.76^c^
GiLPCmTRCiACu*	0.37 ± 0.09^c^	2.56 ± 1.05^d^
GaTCu*PLBNGiCl	0.44 ± 0.05^b^	1.33 ± 0.49^d^
CTch	0.38 ± 0.04^c^	28.45 ± 5.31^b^
Herbs	0.66 ± 0.01^a^	0.6 ± 0^d^

*n*, number of samples analyzed per types of samples collected.

^a‐d^Means with different letters in the same column are significantly different (*p* < .05).

### 3.4 Relation between duration of preservation, water activity, fungal loads, sampling sites, and total aflatoxins levels of SAH studied

**Figure 2:**
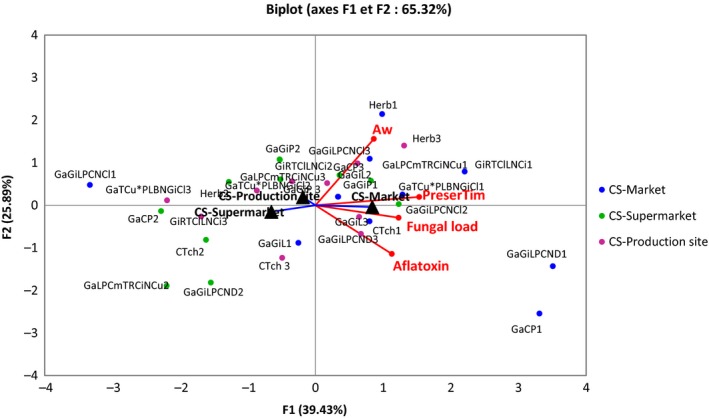
Correspondence Analysis showing linkages between preservation time, water activity, fungal load, and total aflatoxins of SAH samples collected from different sites. *a*_w_, Water activity; PreserTim, Preservation time (mois); Fungal load, Fungal load; Aflatoxin, Aflatoxin Total; CS, Collection site; GaGiP, Garlic–ginger–pepper; GaCP, Garlic–chili–pepper; GaGiL, Garlic–ginger–laurel; GaGiLPCND, Garlic–ginger–nutmeg–dill; GaGiLPCNCl, Garlic–ginger–nutmeg–clove–chili–pepper–laurel; GiRTClLNCi, Ginger–rosemary–thyme–clove–laurel–nutmeg–Cinnamon; GiLPCmTRCiACu, Ginger–laurel–pepper–cumin–thyme–rosemary–cinnamon–anise–curry; GaTCu*PLBNGiCl, Garlicthyme–curry–pepper–laurel–basil–nutmeg–ginger–clove; CTch, Dry chili–nutmeg–cake of peanut–thyme‐salt, maggi; Herbs

Principal component analysis (PCA) was performed to reveal linkage between variables including duration of preservation, water activity, fungal loads, total aflatoxins, and sampling sites of the different SAH powders investigated (Figure 2). The two axes accounted for 65.32% of variations among which 39.43% was explained by the first axis (F1) and 25.89% by the second axis (F2). The preservation time and fungal load were correlated between them and were significantly and positively correlated with the first axe (F1) with correlation coefficients of 0.79 and 0.63, respectively. As shown in Figure 1, most of the SAH samples collected from market were significantly correlated with these two variables except two SAH samples from market. This suggested that, increasing the preservation time of the SAH collected from market could lead to the increase in its fungal load. At the opposite, most of the SAH samples collected from the processing sites and supermarkets were negatively correlated with the first axe (F1) and allowed to assert that the fungal loads of these samples were less influenced by the preservation time. The variable water activity (*a*_w_) was significantly correlated with the second axe F2 (*r* = 0.80). Indeed, the *a*_w_ of the SAH samples collected from markets did not depended to other variables except preservation time which had a slight correlation (*r* = 0.27) with it. It is also noticed that the variables fugal load and total aflatoxins were slightly correlated (*r* = 0.19) between them and this means that the increase in the total aflatoxins may depends on the increase in fungal load in the SAH samples mainly the ones collected from markets, probably due to the environmental conditions. However, the low level of correlation between these two variables (fugal load and total aflatoxins) indicates that the rate of total aflatoxins production in the SAH samples did not directly depends on the fungal load. Thus, all mold is not susceptible to produce aflatoxins and the presence of molds in the spices does not explain directly the presence of mycotoxins. Similar observation has been reported by Nyugen (2007) and Abou (2008).

## 4 CONCLUSION

This study documented the significant occurrence of molds and aflatoxins in SAH powders mostly used by consumers in Benin and neighboring countries. Therefore, the presence of molds and aflatoxins should be controlled during all the food chain including mainly plant growth, processing, and storage steps. One of the preventive measures could be the sorting before processing and the control of water activity in spices and temperature of storage room, which determined the growth of molds. Further investigation related to the identification of molds species involved in the production of aflatoxins in the investigated SAH powders is necessary.
